# miR-3651 Participates in the Growth Cycle of Hepatocellular Carcinoma Cells and Promotes the Malignant Metastasis via the PI3K/AKT/mTOR Signalling Pathway

**DOI:** 10.1155/2022/5744999

**Published:** 2022-09-19

**Authors:** Yawei Liu, Litian Hu, Qinqiang Liu, Jing Ye, Jianping Zhang

**Affiliations:** ^1^Department of General Surgery, Affiliated Drum Tower Hospital of Nanjing University Medical School, Nanjing, Jiangsu 210008, China; ^2^Department of General Surgery, The Second Affiliated Hospital, Nanjing Medical University, Nanjing, Jiangsu 211166, China

## Abstract

**Objective:**

Hepatocellular carcinoma (HCC) shows a growing incidence over the past few years, and clinical efforts are made to search for more effective novel diagnosis and therapy regimen for it to improve its outcome. This study probed into the association of miR-3651 with the PI3K/AKT/mTOR pathway to offer a more detailed reference to the follow-up exploration of novel diagnosis and therapy methods of HCC.

**Methods:**

Totally, 83 patients with HCC treated in our hospital between Apr. 2017 and Aug. 2018, 100 patients with simple liver cirrhosis (LC), and 94 normal persons over the same time span were enrolled, and serum miR-3651 in them was quantified to understand the predictive and prognostic significance of miR-3651 for HCC. In addition, with purchased human HCC cell strains (HepG2), the impacts of miR-3651 on the invasion as well as proliferation of HepG2 were determined using the MTT and Transwell assays, and the PI3K/AKT/mTOR pathway and autophagy-associated proteins in HepG2 were quantified via WT.

**Results:**

Serum miR-3651 was found to be higher in HCC patients than in LC patients and normal persons, and it presented a sensitivity and specificity of 57.14% and 94.00%, respectively, in forecasting the occurrence of HCC in LC patients. The decrease of miR-3651 in HCC patients after therapy was strongly bound up with patients' prognosis, and its increase implied an increased risk of death. In *in vitro* assays, HepG2 presented higher miR-3651 expression than HL-7702, and upregulated miR-3651 intensified the invasion and proliferation of HepG2, while silencing miR-3651 gave rise to opposite results. Additionally, the PI3K/Akt/mTOR pathway in HepG2 presented an obvious activation state, and its activation was further intensified after increase of miR-3651, while its activation was suppressed after silence of miR-3651. Moreover, HepG2 presented notably downregulated autophagy-associated proteins, and the increase of miR-3651 further suppressed the autophagy process, but with the intervention of BEZ235, the impacts of miR-3651 were completely reversed.

**Conclusion:**

miR-3651 intensifies the growth and invasion of HCC cells through activating the PI3K/AKT/mTOR signalling pathway, which is probably a breakthrough in the future diagnosis and therapy of HCC.

## 1. Introduction

Hepatocellular carcinoma (HCC) is one frequently seen malignant tumor worldwide, with a mean annual increase of over 800,000 patients [1]. Its specific pathogenic mechanism is still under investigation, and its primary cause is clinically considered to be bound up with hepatitis B virus infection, liver cirrhosis (LC), fatty liver (and other liver diseases), heredity, and so on [2]. HCC is featured with no symptoms or with only slight atypical symptoms in the early stage usually, and it has reached the middle or late stage, accompanied by different degrees of intrahepatic and extrahepatic metastasis once it shows notable clinical symptoms [3, 4]. According to the survey, HCC in over 70% of HCC patients was in CNLC IIa or above, which is one primary cause for the unfavourable outcome of HCC patients [5]. At the current stage, many feasible clinical therapy approaches are available for HCC, but the 5-year survival rate of HCC patients is still only appropriate 30%-50% [6].

Over the past few years, as a focus of medical research, microRNA has been verified to be a part in many processes such as epigenetics, organ and tissue changes, cell differentiation, and disease development [7]. Among them, miR-3651, firstly discovered in 2014, was confirmed to be a potential biomarker of oral cancer [8, 9]. Over the past few years, Zhu et al. have discovered aberrant increase of miR-3651 in HCC cases through microarray analysis of microRNA [10]. Zhao et al. have confirmed the function of miR-3651 in promoting the growth and invasion of HCC cells through targeting PTEN [11]. The above can confirm the crucial role of miR-3651 in HCC. However, as mentioned by Zhao et al., PTEN primarily impacts cells via the PI3K/Akt/mTOR pathway. Therefore, the associated pathway probably also has a potential connection with miR-3651, but it still requires further confirmation. The association of PI3K/AKT/mTOR signal transduction with miR-3651 in HCC also deserves further analysis. Accordingly, this study probed into the association of miR-3651 with the PI3K/AKT/mTOR pathway to more deeply understand the potential significance of miR-3651 in HCC and offer a more detailed reference to the follow-up exploration of novel diagnosis and therapy methods of HCC.

## 2. Materials and Methods

### 2.1. Patient Information

Totally, 83 HCC patients treated in our hospital between April 2017 and August 2018 and 100 patients with simple LC and 94 normal persons over the same time span were enrolled. The study was performed under strict Declaration of Helsinki and with informed consent forms signed by each participant. The ethics committee of the Affiliated Drum Tower Hospital of Nanjing University Medical School (2019ky002) approved the study. HCC patients were not greatly different from LC patients and normal person in clinical baseline data including age and gender (*P* > 0.05, Table 1).

### 2.2. Inclusion and Exclusion Criteria

HCC patients: patients confirmed with HCC due to LC by biopsy in the Department of Pathology of our hospital, patients in early pathological stage, patients > 18 years old, and those with detailed medical records. Exclude patients with other comorbid benign or malignant tumors, patients with cardiovascular or cerebrovascular diseases or immunodeficiency diseases, patients with dysfunction of crucial organs, patients with poor compliance due to mental disease, pregnant patients, and lactating patients. LC patients: LC was diagnosed after liver biopsy, patients > 18 years old, and those with detailed medical records. The exclusion criteria are the same as above. Normal person: people who have routine physical examination in our hospital, the results of the physical examination are normal, no previous history of major disease, >18 years old, and those with detailed medical records. The exclusion criteria are the same as above.

### 2.3. Acquisition of Clinical Specimens

Fasting venous blood (5 mL) acquired from each HCC patient at admission and 6 hours after radical operation were put in coagulation-promoting tubes and let to stand at indoor temperature, followed by centrifugation for acquiring serum that was saved in a refrigerator (-80°C) for later analysis. A 2-year follow-up was conducted to every HCC patient, on which the overall survival rate was recorded.

### 2.4. Cell Culture and Treatment

HepG2 and HL-7702 offered by ATCC were subjected to incubation (5% CO_2_, 37°C) in 10% fetal bovine serum-contained DMEM.

### 2.5. Cell Transfection

miR-3651 mimic sequence (3651-mimics group), inhibitor sequence (3651-inhibition group), and negative control (3651-NC group) were transfected into HepG2 under the instructions of Lipofectamine 2000 kit (Thermo Fisher Scientific, the States), followed by quantification of miR-3651 by PCR to verify the transfection success rate. Additionally, 5 mmol/L stock solution was prepared by dissolving the PI3K/AKT/mTOR signalling pathway inhibitor BEZ235 into sterile DMSO, followed by dilution to 0.1 *μ*mol/L with blood-free medium, and the prepared solution was used to intervene with HCC (BEZ235 group). A control group was set by culturing normal HCC cells with the same amount of normal saline.

### 2.6. qRT-PCR

Total RNA acquired via the Trizol method was treated by reverse transcription via a reverse transcription kit (Thermo Fisher Scientific, the States) under kit instructions to acquire cDNA that was then treated by amplification reaction. Reaction conditions: GenScript Biotechnology Co., Ltd. designed and constructed the primer sequences (Table 2). Gene expression was calculated via the 2^-*ΔΔ*Ct^ method (internal reference: U6).

### 2.7. Determination of Cell Viability

Transfected cells were transferred to 96-well plates (4 × 10^5^ cells/well), and each group was provided with 3 duplicate wells. Then, the plates were subjected to routine culture with culture medium, followed by liquid replacement and addition of 20 *μ*L MTT solution (Abcam, China) at 24, 48, and 72 h for incubation. The supernatant was removed after 4 h continuous incubation, followed by addition of formazan solution (Abcam, China) to stop the reaction. A microplate reader was adopted for determining the optical density (OD) at 490 nm, on which cell growth curves were drawn.

### 2.8. Determination of Cell Invasion Ability

Diluted Matrigel along with resuspended cells was placed in the upper compartment of the Transwell chamber and 10% serum-contained DMEM to the lower compartment. After 24 h, the membrane-penetrating cells were treated by immobilization and staining and then counted under the microscope.

### 2.9. Quantification of Proteins

Cell lysate was adopted for lysing cells that were then treated by centrifugation, and then, BCA (Beyotime Biotechnology, China) was adopted for quantification of the total protein. Total protein (50 *μ*g) was treated by SDS-PAGE and placed on a membrane, followed by immersion in defatted milk, addition of I antibody, and overnight incubation (4°C). II antibody was put into it after TBST washing the next day, and ECL was developed after 1 h incubation. The protein expression was calculated (internal reference: GAPDH). Antibodies were all offered by Thermo Fisher Scientific (the States).

### 2.10. Statistical Analyses

This study adopted SPSS 22.0 for statistical analyses. Intergroup comparison of counting data [*n* (%)] was conducted via the chi-square test, and intergroup comparison of measurement data (^−^*χ* ± s) was performed via the *t*-test, one-way ANOVA, and post hoc LSD test. ROC curves were drawn for analyzing the predictive value, and the Kaplan-Meier method was adopted for calculating the survival rate and the log-rank test for comparing it. *P* < 0.05 implies a notable difference.

## 3. Results

### 3.1. miR-3651 Increases in HCC

**Figure 1:**
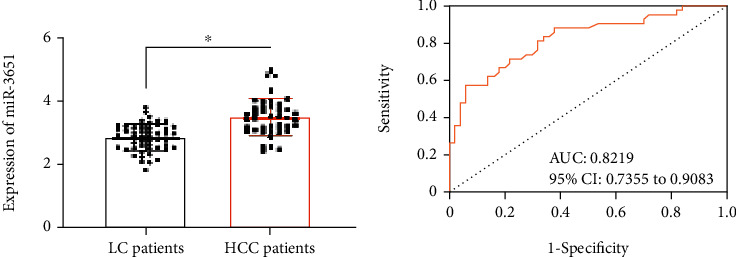
miR-3651 expression in HCC. (a) Comparison of miR-3651 levels in serum of HCC patients (*n* = 83), LC patients (*n* = 100), and normal persons (*n* = 94). (b) ROC curve of miR-3651 for forecasting the occurrence of HCC in LC patients. Comparison of two groups ^∗^*P* < 0.05.

According to PCR assay results, HCC patients showed higher serum miR-3651 than LC patients and normal person (3.53 ± 0.59*vs.*2.87 ± 0.49*vs.*1.78 ± 0.44, *P* < 0.05, Figure 1(a)). According to ROC curve-based analysis, under the area under the curve (AUC) of 0.8219, serum miR-3651 > 3.41 had a sensitivity and specificity of 59.04% and 92.00%, respectively, in forecasting the occurrence of HCC in LC patients (*P* < 0.05, Figure 1(b)).

### 3.2. High miR-3651 Expression Implies Unfavourable Prognosis of HCC

**Figure 2:**
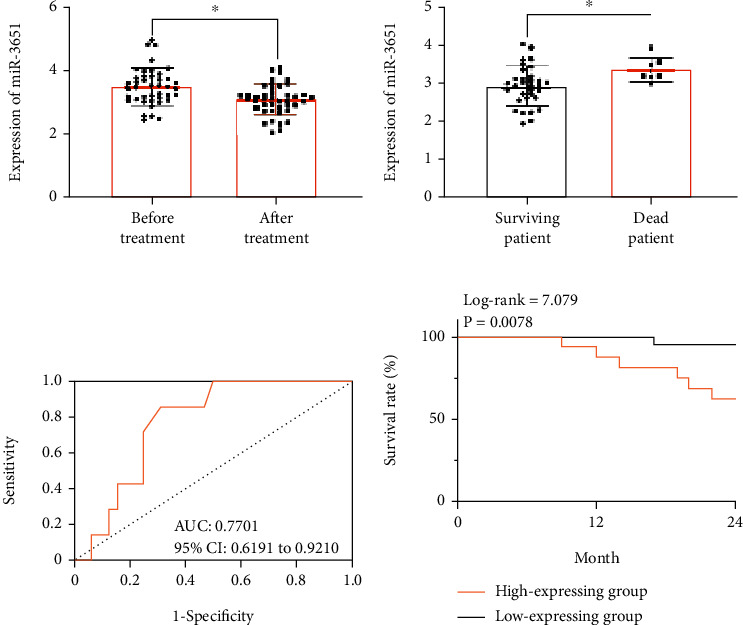
Prognostic significance of miR-3651. (a) Serum miR-3651 in HCC patients before and after therapy (*n* = 83). (b) Comparison of miR-3651 expression between the patients who died finally (*n* = 17) and those who survived (*n* = 61). (c) ROC curve of miR-3651 for forecasting the death of HCC patients within 2 years after therapy. (d) Survival curves of the high- (*n* = 33) and low-expression groups (*n* = 45). Comparison of two groups ^∗^*P* < 0.05.

After therapy, HCC patients presented serum miR-3651 of 3.00 ± 0.48, notably lower than that at admission (*P* < 0.05, Figure 2(a)). In terms of follow-up, 78 HCC patients were successfully tracked, of which 17 patients died, so the overall 2-year mortality was 21.79%. Patients who died eventually presented serum miR-3651 of 3.41 ± 0.32 after therapy, notably higher than that of those who survived finally after therapy (*P* < 0.05, Figure 2(b)). According to ROC curve-based analysis, under the AUC of 0.7701, serum miR-3651 > 3.21 had a sensitivity and a specificity of 94.12% and 70.49%, respectively, in forecasting the death of HCC patients within 2 years (*P* < 0.05, Figure 2(c)). Finally, based on the cutoff value, the patients were grouped into high-expression groups (miR-3651 > 3.12 after therapy for the former, *n* = 33) and low-expression groups (miR-3651 ≤ 3.12 after therapy for the former, *n* = 45). According to comparison of the two groups' survival curves, the high-expression group presented a notably higher mortality than the other (*P* < 0.05, Figure 2(d)).

### 3.3. Upregulating miR-3651 Can Intensify the Invasion and Proliferation of HCC Cells

**Figure 3:**
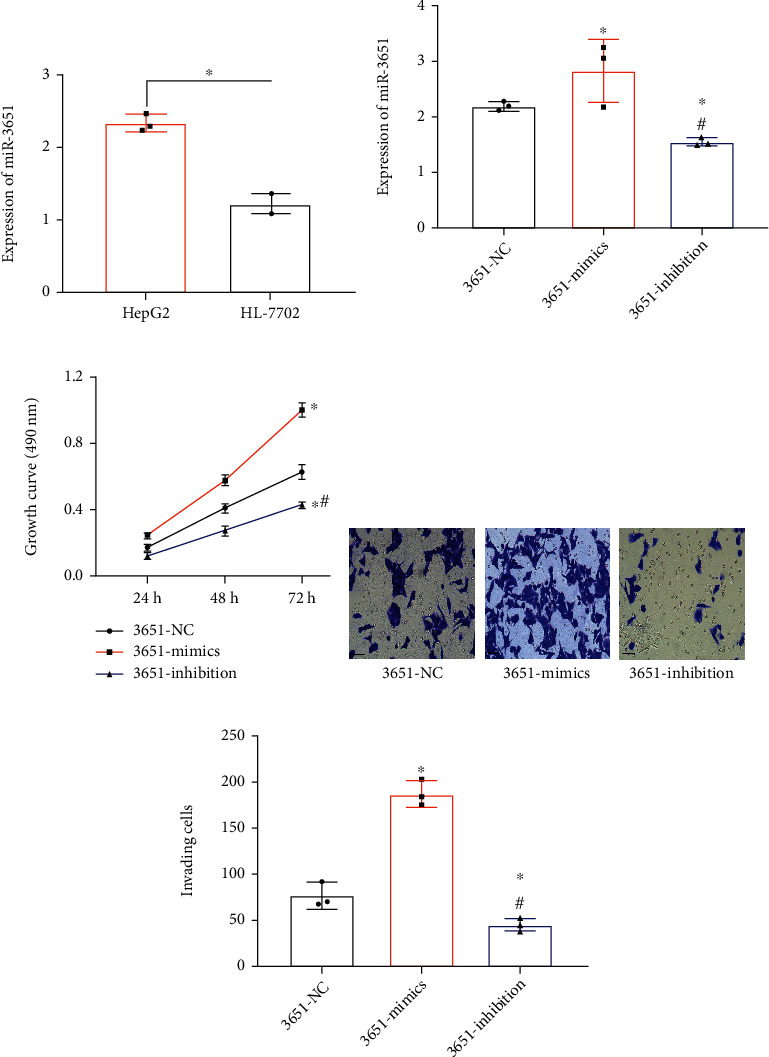
Impacts of miR-3651 on HCC cell viability (*n* = 3). (a) miR-3651 expression in HepG2 and HL-7702. Comparison of two groups ^∗^*P* < 0.05. (b) PCR verified the success rate of transfection. (c) Growth curve of HepG2. (d) Staining of invasion cells (×200). (e) Counting of invasive cells. Compared with the 3651-NC group ^∗^*P* < 0.05. Compared with the 3651-mimics group ^#^*P* < 0.05.

In *in vitro* assays, HepG2 showed higher miR-3651 expression than HL-7702 (2.33 ± 0.12*vs.*1.21 ± 0.14, *P* < 0.05, Figure 3(a)). After transfection, the 3651-mimic group presented the highest miR-3651 expression (2.83 ± 0.57), and the 3651-inhibition group presented lower miR-3651 expression than the 3651-NC group (1.55 ± 0.07*vs.*2.20 ± 0.08, *P* < 0.05, Figure 3(b)), which verified the success of transfection. According to the results of MTT assay, the largest 72 h OD was found in the 3651-mimics group, followed by the 3651-NC group and the 3651-inhibition group (1.00 ± 0.04 > 0.63 ± 0.05 > 0.43 ± 0.02, all *P* < 0.05, Figure 3(c)). According to the Transwell assay (Figure 3(d)), the largest number of invasive cells was found in the 3651-mimics group, followed by the 3651-NC group and the 3651-inhibition group (187.67 ± 13.87 > 78.33 ± 13.65 > 46.00 ± 6.56, all *P* < 0.05, Figure 3(e)).

### 3.4. miR-3651 Can Activate the PI3K/AKT/mTOR Signalling Pathway

**Figure 4:**
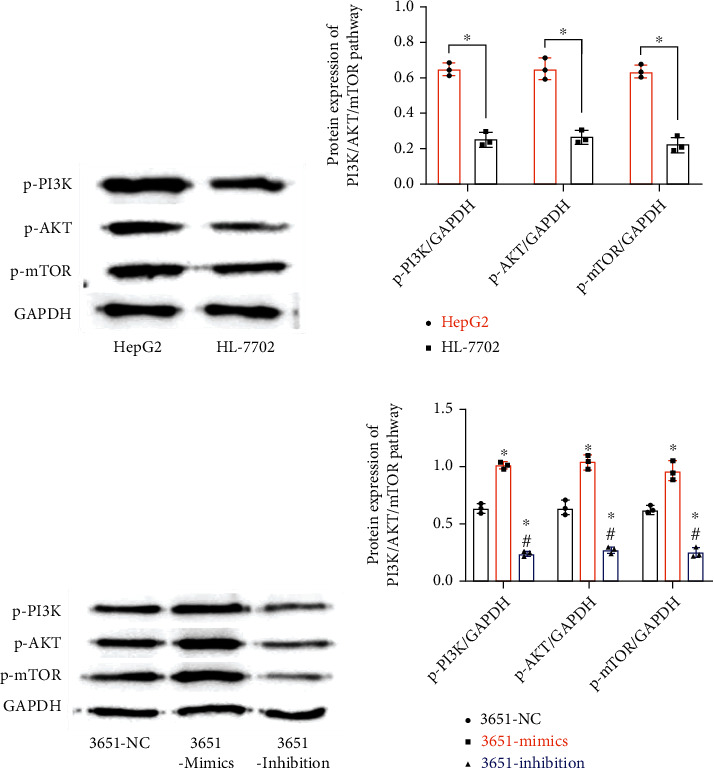
Impacts of miR-3651 on the PI3K/AKT/mTOR signalling pathway in HCC cells (*n* = 3). (a) Western blot image. (b) Expression of PI3K/AKT/mTOR pathway-associated proteins in HepG2 and HL-7702. Comparison of two groups ^∗^*P* < 0.05. (c) Western blot image. (d) Expression of the PI3K/AKT/mTOR pathway in HepG2. Compared with the 3651-NC group ^∗^*P* < 0.05. Compared with the 3651-mimics group ^#^*P* < 0.05.

PI3K/AKT/mTOR pathway-associated proteins in HepG2 and HL-7702 were quantified (Figure 4(a)). As a result, HepG2 presented expression of P-PI3K, P-Akt, and P-mTOR proteins of 0.64 ± 0.04, 0.65 ± 0.06, and 0.63 ± 0.04, respectively, higher than that in HL-7702 (*P* < 0.05, Figure 4(b)). Subsequently, the expression of PI3K/AKT/mTOR pathway-related proteins in HepG2 after transfection was detected (Figure 4(c)); the 3651 mimics presented p-PI3K protein expression of 1.01 ± 0.03, p-AKT expression of 1.04 ± 0.07, and p-mTOR expression of 0.97 ± 0.09, which were all the highest among the three groups. The 3651-inhibition group presented p-PI3K protein expression of 0.24 ± 0.02, p-AKT expression of 0.27 ± 0.03, and p-mTOR expression of 0.25 ± 0.04, lower than those in the 3651-NC group (*P* < 0.05, Figure 4(d)).

### 3.5. Suppressing the PI3K/AKT/mTOR Signalling Pathway Can Weaken the Proliferation and Invasion of HCC Cells

**Figure 5:**
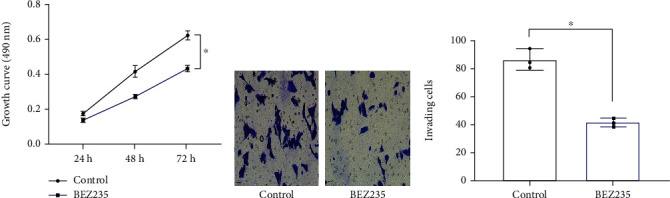
Impacts of the PI3K/AKT/mTOR signalling pathway on HCC cell viability (*n* = 3). (a) Growth curve of HepG2. (b) Staining of invasion cells (×200). (c) Counting of invasive cells. Comparison of two groups ^∗^*P* < 0.05.

The BEZ235 group showed a notably lower 72 h OD than the control group (0.43 ± 0.02*vs.*0.62 ± 0.03, *P* < 0.05, Figure 5(a)). The Transwell assay results (Figure 5(b)) revealed notably less invasive cells in the BEZ235 group than in the control group (42.00 ± 3.00*vs.*86.67 ± 7.37, *P* < 0.05, Figure 5(c)).

### 3.6. The Impacts of miR-3651 on the Autophagy of HCC Cells Can Be Reversed by the PI3K/AKT/mTOR Pathway

**Figure 6:**
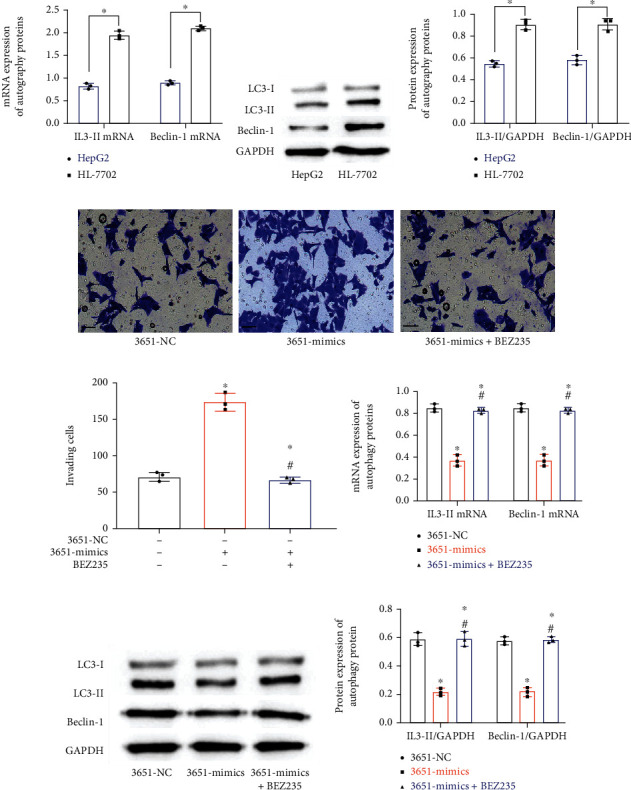
miR-3651 impacts the autophagy of HCC cells via the PI3K/AKT/mTOR pathway (*n* = 3). (a) Quantification of autophagy-associated proteins in HepG2 and HL-7702 by PCR. (b) WT profile. (c) Quantification of autophagy-associated proteins in HepG2 and HL-7702 by WT. Comparison of two groups ^∗^*P* < 0.05. (d) Staining of invasion cells (×200). (e) Counting of invasive cells. (f) Quantification of autophagy-associated proteins by PCR under the intervention of miR-3651 and PI3K/AKT/mTOR. (g) Western blot image. (h) Quantification of autophagy-associated proteins by WT under the intervention of miR-3651 and PI3K/AKT/mTOR. Compared with the 3651-NC group ^∗^*P* < 0.05. Compared with the 3651-mimics group ^#^*P* < 0.05.

The PCR assay revealed lower LC3-II mRNA and Beclin-1 mRNA expression in HepG2 than in HL-7702 (0.81 ± 0.06*vs.*1.94 ± 0.09; 0.88 ± 0.04*vs.*2.10 ± 0.04; *P* < 0.05, Figure 6(a)). According to the Western blot results (Figure 6(b)), the expression of LC3-II and Beclin-1 proteins in HepG2 was 0.55 ± 0.03 and 0.58 ± 0.04, respectively, also notably lower than that in HL-7702 (*P* < 0.05, Figure 6(c)). Afterwards, HepG2 treated with miR-3651 mimics was cultured with BEZ235, and separate 3651-mimics group and 3651-NC group were set up. First, through Transwell experiments (Figure 6(d)), we found that the number of invasive cells in the 3651-mimics+BEZ235 group was 67.67 ± 3.51, and there was no difference in the number of invasive cells in the 3651-NC group (72.00 ± 6.08), which was lower than that of the 3651-mimics group (*P* < 0.05, Figure 6(e)). According to the results, the 3651-mimics+BEZ235 group was not greatly different from the 3651-NC group in LC3-II and Beclin-1 mRNA levels (0.82 ± 0.03*vs.*0.85 ± 0.04; 0.85 ± 0.04*vs.*0.89 ± 0.03), but showed notably lower levels of them than the 3651-mimics group (*P* < 0.05, Figure 6(f)). Similarly, according to the Western blot results (Figure 6(g)), the 3651-mimics+BEZ235 group was also not greatly different from the 3651-NC group in LC3-II and Beclin-1 protein levels (0.59 ± 0.05*vs.*0.59 ± 0.05; 0.58 ± 0.03*vs.*0.57 ± 0.03), but showed notably lower levels of them than the 3651-mimic group (*P* < 0.05, Figure 6(h)).

## 4. Discussion

HCC is a high-risk disease that endangers the lives of millions of patients worldwide. Finding a more effective and accurate diagnosis and therapy means as soon as possible is the only solution to ensure the health of patients with it [12]. Over the past few years, the application of microRNA in tumor diseases has captured attention, which has laid a promising direction for the future molecular gene targeted therapy [13]. This study more deeply probed into the association of miR-3651 with HCC, which could offer more accurate and effective reference and guidance for the follow-up research.

Prior research confirmed aberrant miR-3651 expression in HCC cases, and our study also verified it. In our study, HCC patients presented notably higher serum miR-3651 expression than LC patients, suggesting high miR-3651 expression in HCC cases, which was in agreement with the prior research results [14, 15]. Our study selected patients with simple LC as controls for ROC analysis, because LC has an enormous risk of pathological changes as the most crucial primary cause of HCC [16]. HCC is occult at the early stage, and a reliable means to monitor the possibility of HCC in LC patients is still under investigation [17]. In our study, miR-3651 demonstrated an excellent performance in forecasting the development of LC into HCC, which once again verified the crucial reference significance of miR-3651 for the future assessment of HCC. In addition, HCC patients showed a notable decrease in miR-3651 level after therapy, which verified a strong association of miR-3651 with the changes of HCC. Moreover, generally higher miR-3651 expression was found in patients who died after therapy, and miR-3651 presented a sensitivity of 85.71% in forecasting the death of patients, which indicated the great potential of miR-3651 to be an indicator for evaluating the prognosis of HCC. Barzago et al. have pointed out the potential of miR-3651 to be a prognostic marker of infection-related inflammatory diseases, which once again emphasized the crucial association of miR-3651 with tumor diseases [18]. According to the survival curves in our study, the increase of miR-3651 directly indicated the increased risk of death of HCC patients, which supported our above results and viewpoints.

To sum up, we have preliminarily discussed the clinical application of miR-3651 in HCC. Prior research has verified the ability of miR-3651 to speed up the proliferation and invasion of HCC cells and the possible association of the pathway with PTEN. Accordingly, for the purpose of verifying the mechanism of miR-3651 in HCC, we also carried out *in vitro* assays. First of all, similar to the above results, the *in vitro* assays also revealed an increase of miR-3651 expression in HepG2. Similar to prior research results, the biological behaviour assays revealed that increasing miR-3651 could intensify the proliferation and invasion of HepG2, while silencing it could give rise to opposite results [19]. The repeated confirmation with various HCC cell strains showed the ability of overexpressed miR-3651 to enhance the activation of HCC cells. As we mentioned above, the PI3K/Akt/mTOR signalling pathway has been confirmed to be implicated in the development of many tumor diseases as the main downstream transduction pathway of PTEN [20]. Our study also found the obvious activation state of the PI3K/Akt/mTOR pathway in HepG2, which confirmed the association of PI3K/Akt/mTOR pathway with HCC. Moreover, the activation state of the pathway was further enhanced after the increase of miR-3651, while the activation state was suppressed after silence of miR-3651, suggesting the regulatory role of miR-3651 to the PI3K/Akt/mTOR pathway in HCC. Under the intervention of BEZ235, the growth and invasion activities of HepG2 cells were greatly suppressed, which indicated that suppression of the PI3K/Akt/mTOR pathway could impact the viability of HCC cells, which was consistent with prior research results [21]. In addition, the PI3K/Akt/mTOR pathway showed a strong correlation with the autophagy ability of tumor cells in our study, so we also detected the autophagy-associated proteins in HepG2 and found notable decrease of autophagy ability in HepG2. The increase of miR-3651 further weakened the autophagy process, and the impact of miR-3651 was completely reversed with the intervention of BEZ235. It follows from the above results that miR-3651 can regulate the viability and autophagy of HCC cells through mediating the PI3K/Akt/mTOR pathway, which lays a foundation for the possible targeted therapy of HCC in the future. We believe that the application of miR-3651 can not only serve as a reference for the diagnosis of HCC but also become a new treatment plan for HCC, thus providing a more reliable guarantee for the life safety of patients.

Of course, we still need to confirm the effect of miR-3651 on living tumors through animal experiments. However, due to the limited experimental conditions, we cannot carry out animal experiments in this experiment, and we will add this in the future. In addition, we could further confirm the effect of miR-3651 on HCC cells through experiments such as cell cloning, scratching, and flow cytometry. However, due to limited funding, we also did not conduct these experiments. Finally, the effect of miR-3651 on HCC cells may be not only through the PI3K/AKT/mTOR signalling pathway, which also requires us to conduct more studies for analysis. In the future, we need to include more patient data to confirm the exact clinical significance of miR-3651 in HCC and conduct a more in-depth and comprehensive analysis of the action pathway of miR-3651 in HCC to obtain more effective results for clinical reference.

To sum up, miR-3651 intensifies the growth and invasion of HCC cells through activating the PI3K/AKT/mTOR signalling pathway, which is probably a breakthrough in the future diagnosis and therapy of HCC, so it is worthy of further explorations.

## Figures and Tables

**Table 1:** Comparison of baseline data of HCC patients, LC patients, and normal people.

	Normal person (*n* = 94)	LC patients (*n* = 100)	HCC patients (*n* = 83)	*t* or *χ*^2^	*P*
Age	56.68 ± 5.10	56.12 ± 4.71	55.60 ± 5.11	1.046	0.353
BMI (kg/m^2^)	23.02 ± 2.30	23.39 ± 1.78	23.05 ± 1.97^∗^	0.992	0.372
Gender				1.112	0.573
Male	62	70	52		
Female	32	30	31		
Family history of illness				1.003	0.606
Have	9	12	12		
None	85	88	71		
Smoking				0.422	0.810
Yes	38	45	36		
No	56	55	47		
Drinking				1.400	0.497
Yes	30	37	24		
No	64	63	59		
Place of residence				2.089	0.352
Urban	67	80	64		
Rural	27	20	19		
Child-Pugh grading				2.691	0.260
A	—	36	21		
B	—	50	46		
C	—	14	16		
TNM staging				—	—
I	—	—	39		
II	—	—	44		

**Table 2:** Primer sequences.

	F (5′-3′)	R (5′-3′)
miR-3651	CCGGTCGCTGGTACATGAC	CTCAACTGGTGTCGTGGAGTC
U6	CTCGCTTCGGCAGCACAT	AACGCTTCACGAATTTGCGT

## Data Availability

The data used to support the findings of this study are available from the corresponding author upon request.
